# 

**Published:** 1889-01

**Authors:** 


### CONTENTS.

COMMUNICATION.

Treatment of Temporary Teeth........ 5
The Third Molar........ 8
What Varieties in Number are Found in the Teeth of Mammals........10
Brief Description of the Shedding of Temporary Teeth, as to Time, Order and Process........12
Variations of Using Teeth in Various Animals........15
Aluminum........17
Anæsthetic in Dental Practice........21

Monthly Digest........31

Protection........42
By-Laws of the Dental Protective Association of the United States........45
Chicago Dental Society Meeting........48

EDITORIAL.

1889........52
1856—1889........54
A Change........55
Obituary........55
Dentists' Apportionment Book........56

